# Influence of Physicochemical Factors on Adsorption of Ten *Shigella flexneri* Phages

**DOI:** 10.3390/v14122815

**Published:** 2022-12-16

**Authors:** David Tomat, Virginia Aquili, Cecilia Casabonne, Andrea Quiberoni

**Affiliations:** 1 Facultad de Ciencias Bioquímicas y Farmacéuticas, Universidad Nacional de Rosario, Suipacha 531, Rosario 2000, Argentina; 2 Instituto de Lactología Industrial (UNL-CONICET), Facultad de Ingeniería Química, Santiago del Estero 2829, Santa Fe 3000, Argentina

**Keywords:** bacteriophage, adsorption, *Shigella flexneri*, food safety

## Abstract

Bacterial viruses known as bacteriophages have been demonstrated to be effective in killing foodborne pathogens such as *Shigella flexneri*. Adsorption is the first step in the phage–host interaction. In the present work, 10 *Shigella* phages were used to characterize the adsorption process on *Shigella flexneri* ATCC12022 in several physicochemical conditions related to food and in a food matrix. One-step growth curves were drawn for all the *Shigella*-phages evaluated. Furthermore, the adsorption rate for each of the 10 phages was determined. In addition, the influence of temperature, Na^+^, Mg^2+^, pH, sucrose and glycerol on phage adsorption was investigated. Two phages (Shi22 and Shi30) showed higher burst sizes values (67 and 64 PFU cell^−1^, respectively) and burst times of 25 min to 30 min, while the other eight phages exhibited burst sizes ranging from 14 to 17 PFU cell^−1^ with slower burst times. Furthermore, most phages achieved a high adsorption rate, and the adsorption constants (*k*) ranged from ~10^−9^ to 10^−10^ mL min^−1^. Regarding the influence of temperature, cations and pH, a high or moderate percentage of adsorption was observed for most of the phages evaluated. The adsorption decreased at increasing concentrations of Na^+^, sucrose and glycerol, although at different levels, since adsorption was more affected by sucrose than by glycerol and Na^+^ for most phages. The adsorption obtained in Triptein soy broth (TSB) for most of the phages/strain systems evaluated was moderate or high, as well as those observed in a food matrix. Thus, our phages could potentially be used to improve food safety under a wide range of environmental conditions against foodborne pathogens.

## 1. Introduction

The foodborne pathogen *Shigella flexneri* is most frequently associated with shigellosis outbreaks in developing countries [1,2,3] such as Argentina [4] and related to the ingestion of contaminated water and food [5]. Shigellosis is recognized as an important cause of children’s mortality in Argentina [6] and is the most important cause of bloody diarrhea worldwide [7].

An increasing antibiotic resistance was observed in recent years [8] among *Shigella* populations [9]. In addition, *Shigella* is spread by direct contact with an infected person, or by eating contaminated food or drinking contaminated water [10]. Therefore, there is a need to develop new strategies in order to fight against foodborne pathogens resistant to antibiotics, as designated for *Shigella* strains [11]. Bacteriophages are the natural enemies of bacteria and may be used as a preventive technology in order to reduce the incidence of *Shigella* infections and enhance food safety. Unlike antibiotics, phages are very specific viruses that kill only the targeted bacteria without affecting any other beneficial bacteria present in food. Furthermore, phages have been generally recognized as having safe (GRAS) status, as granted by the Food and Drug Administration.

Adsorption of phages to the cell surface is the first step in the phage life cycle and determines the whole infection process [12]. Abiotic conditions such as pH and temperature are known to play an important role in the phage lifecycle. A better understanding of phage–host interactions in different abiotic conditions can lead to more effective biocontrol treatments. Although studies on the isolation or characterization of *Shigella* phages have been conducted [13,14], those focusing on adsorption of *S*. *flexneri* phages have hardly been documented.

In our previous study, 10 phages lytic against *S*. *flexneri* strains were isolated and characterized by their host range and challenge assays, including stability tests under various stress conditions [14]. Thus, the aim of the present work was to characterize the phage–host interaction through parameters of the phage cycle and adsorption constants (*k*), and to evaluate the influence of physicochemical conditions relevant to food processing on phage adsorption to improve the efficiency of application on the food matrix. To our knowledge, this is the first study focused on evaluating adsorption of *S*. *flexneri* phages at several abiotic conditions, such as varying temperature and pH as well as different concentrations of Na^+^, Mg^2+^ and sucrose.

## 2. Materials and Methods

### 2.1. Bacterial Strains and Phages

ATCC12022, a *Shigella flexneri* strain belonging to serotype 2b, was used in adsorption studies and as a host strain to propagate bacteriophages. Stocks at −80 °C in Triptein soy broth (TSB; pancreatic digest of casein 17 g L^−1^, papaic digest of soybean 3 g L^−1^, sodium chloride 5 g L^−1^, dipotassium phosphate 2.5 g L^−1^, glucose 2.5 g L^−1^ supplemented with 15% *v/v* glycerol) were reactivated overnight in TSB at 37 °C for assays with *Shigella* phages.

In this study, 10 bacteriophages, namely AShi, Shi3, Shi22, Shi30, Shi33, Shi34, Shi40, Shi88, Shi93 and Shi113, previously isolated from 114 stool samples [14], were used to evaluate their growth curve and adsorption characteristics.

### 2.2. One Step Growth Curve

A one-step growth analysis was carried out for the 10 *Shigella* phages evaluated. Briefly, ATCC12022 cells were grown (optical density at 600 nm-OD_600nm_ -of 0.1), then harvested by centrifugation (1 min at 14,000× *g*), and the pellet obtained was resuspended (500 μL of TSB) and mixed with 500 μL of each phage evaluated (multiplicity of infection-MOI = 0.01). The ATCC12022-phage mixture was incubated (2 min at 37 °C) and then centrifuged (1 min at 14,000× *g*) to remove free phages. The pellet was resuspended in 10 mL of fresh TSB and incubated at 37 °C. Phages were enumerated every 10 min by the double-layer plaque technique [15]. Plaques were incubated for 18 h at 37 °C, and the plaque forming units (PFU) were counted to calculate the burst size for each phage by the equation described by Wang [16].

### 2.3. Adsorption Studies

Adsorption studies of phages (100 µL, 10^5^ PFU) on bacteria (500 µL, OD_600nm_ = 1.0) were conducted using a MOI of 0.001 in order to achieve the maximum adsorption possible. Following incubation at 25 °C for 10 min, suspensions were centrifuged for 5 min at 14,000× *g*. Then, free phages (supernatant) were enumerated by the double-layer plaque technique [15]. Non-adsorbing control (phages-10^5^ PFU in TSB without cells incubated at 25 °C for 10 min, then centrifuged for 5 min at 14,000× *g*-and enumerated) was carried out for each phage evaluated (100% free phage).

#### 2.3.1. Adsorption Rate

Assays were conducted as described in Section 2.3 (25 °C; MOI = 0.001) to evaluate the phage adsorption rate. A non-adsorbing control (TSB without cells) was performed for each phage evaluated for setting the value of time 0 min (100% free phage). Then, samples were taken every 2 or 3 min (up to 17 min), centrifuged (1 min at 14,000× *g*) and free phages in the supernatant were enumerated by the double-layer plaque technique [15]. Finally, the adsorption constant *k* for each phage was calculated as previously described [17].

#### 2.3.2. Influence of Physicochemical Factors

Adsorption of *Shigella* phages on ATCC12022 cells was evaluated under several conditions. Temperature: 4, 25 (control), 37 and 50 °C. Cations: NaCl (TSB, 1, 2, 4, 10 and 20 g dL^−1^) or MgSO_4_ (1, 5 and 10 mmol L^−1^). pH: 4, 7 (control) and 10. Sucrose: 5, 10 and 20 g dL^−1^. Glycerol: 5, 10, 20 and 30 g dL^−1^. Cells were grown in TSB for 18 h at 37 °C at each condition tested for adsorption assays. ATCC12022 was inhibited at 10 and 20 g dL^−1^ of NaCl, at pH 4 and 10 and at 20 and 30 g dL^−1^ of glycerol. Thus, cells of ATCC12022 were previously grown in TSB (18 h at 37 °C) and then centrifuged and resuspended at those salinities, with pH and glycerol values used to carry out experiments at the inhibitory conditions. Results were expressed as percentages of adsorption after 10 min of phage-cells interaction regarding non-adsorbing controls.

#### 2.3.3. Adsorption in Meat

Adsorption studies of phages (10 µL, 10^5^ PFU) on bacteria (10 µL, OD_600nm_ = 1.0) in meat were conducted using an MOI of 0.001. Beef was aseptically cut into pieces (1 cm^2^; pH 5.6), placed in petri dishes and pre-equilibrated to 25 °C. Ten μL of ATCC12022 suspension were pipetted onto the surface of the meat sample and allowed to attach for ten minutes at room temperature. Next, 10 μL of each phage were pipetted on the meat surface. Following incubation at 25 °C for 10 min, meat pieces were transferred to a sterile tube with 1 mL of TSB and mixed for 1 min. A sample (0.6 mL) of the liquid portion was transferred to a sterile eppendorf and centrifuged for 5 min at 14,000× *g*. Then, free phages (supernatant) were enumerated by the double-layer plaque technique [15]. Nonadsorbing control (meat without cells) was carried out for each phage evaluated (100% free phage).

### 2.4. Statistical Analysis

Three independent experiments (biological replicates) were carried out. The mean values of treatments and controls were compared using the student’s *t* test at *p* < 0.05.

## 3. Results

### 3.1. One Step Growth Curve Experiment

Parameters such as burst size and latent period were investigated through the one-step growth curve. Although a similar behavior was found for all the phages evaluated, different values were observed (Figure S1). Studies showed that the latent period and burst size of phage Shi22 were 25 min and 67 PFU cell^−1^, respectively. In addition, the latent period was followed by a burst period of about 25 min with an average burst time of 40 min. Shi30 showed a similar behavior to Shi22 regarding the burst size (64 PFU cell^−1^). Moreover, Shi30 revealed the longer latent period (30 min) of all the phages tested.

Regarding the other phages evaluated, all of them showed a relatively low burst size, presenting values ranging from 14 to 17 PFU cell^−1^. Thus, experiments revealed two groups of phages: two phages with a large burst size, namely Shi22 and Shi30, and eight phages with a low burst size, namely AShi, Shi3, Shi33, Shi34, Shi40, Shi88, Shi93 and Shi113 (Table 1). Furthermore, phages with a large burst size presented a slightly higher latent period (25 min to 30 min) than those with lower values (15 min to 25 min).

### 3.2. Adsorption Studies

#### 3.2.1. Adsorption Rate

**Figure 1:**
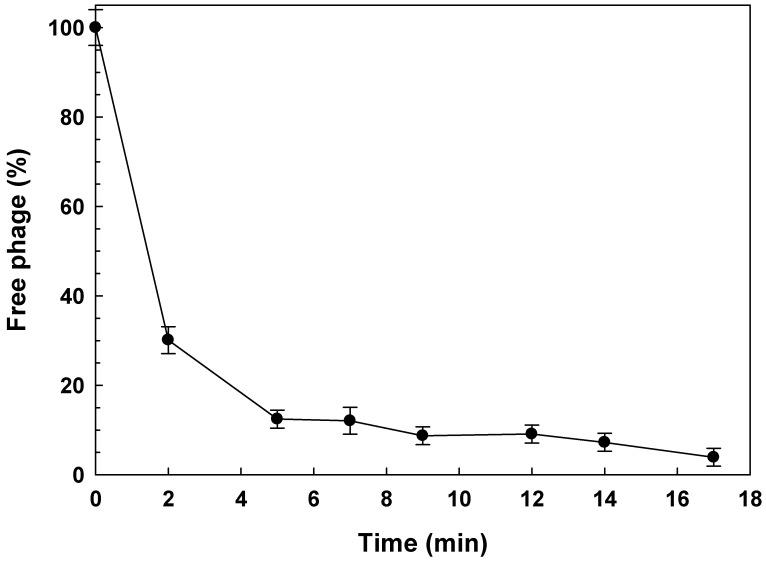
Phage Shi22 adsorption rate in TSB at 25 °C on ATCC12022 viable cells. A similar behavior was found for all the phages evaluated. Values are the mean ± standard deviation (error bars) of three determinations.

Adsorption experiments revealed that our *Shigella* phages adsorbed differentially to ATCC12022 cells. Figure 1 shows a representative adsorption curve (phage Shi22). Four phages showed that a 70% or more of their particles adsorbed within 5 min (AShi, Shi3, Shi22 and Shi113), while, for the other six phages tested, the adsorption rate ranged from 21 to 56% after a 5-min incubation (MOI = 0.001), namely Shi30, Shi33, Shi34, Shi40, Shi88 and Shi93 (Table 1). At the end (17 min) of each experiment, six phages significantly increased (AShi, Shi22, Shi30, Shi34, Shi40 and Shi93) and the other three slightly increased (Shi3, Shi33 and Shi88) their amount of particles adsorbed. One phage presented an adsorption that remained unchanged (Shi113) within 17 min (Table 1).

In this work, the adsorption rate constant *k* ranged from 4.64 × 10^−10^ mL min^−1^ to 4.16 × 10^−9^ mL min^−1^ after a 5-min phage–cell interaction at 25 °C (MOI = 0.001) (Table 1). Specifically, most phages showed values of *k* within the same order of magnitude (AShi, Shi3, Shi22, Shi30, Shi33, Shi34, Shi88, Shi93 and Shi113), while Shi40 presented the lower *k* value (4.64 × 10^−10^ mL min^−1^) of all the phages tested. In addition, phages with the highest *k* values (AShi and Shi22) were the only ones that achieved a high adsorption rate (>96%) at the end of the experiments (17 min).

#### 3.2.2. Influence of Temperature

**Figure 2:**
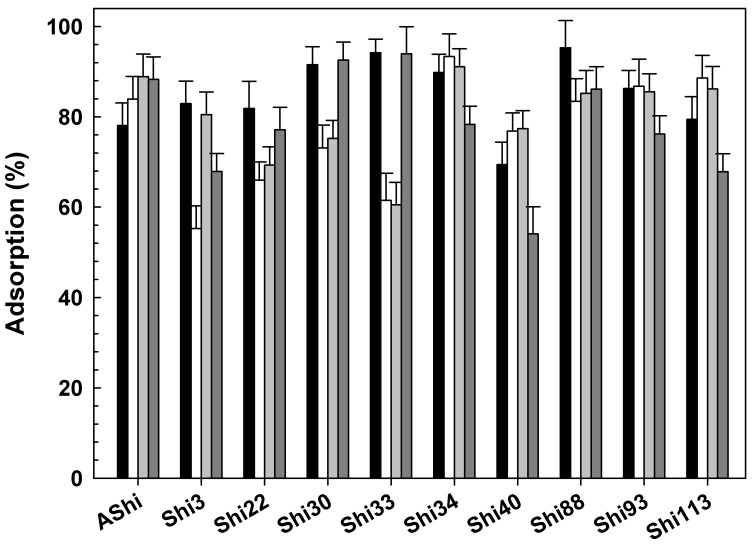
Phage adsorption in TSB at 4 °C (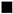), 25 °C (☐; control), 37 °C (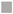) and 50 °C (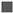) on ATCC12022 viable cells. Values are the mean ± standard deviation (error bars) of three determinations.

The effect of temperature on phage adsorption is shown in Figure 2. Results showed that phages can be efficiently absorbed even at the highest and lowest temperature assayed. At moderate temperatures (25 and 37 °C), phage adsorption showed no significant differences in nine of the ten phages tested, while Shi3 proved a higher adsorption at 37 °C than at 25 °C. When comparing adsorption between extreme temperatures (4 and 50 °C), six phages (Shi3, Shi34, Shi40, Shi88, Shi93 and Shi113) presented a higher adsorption value at 4 °C than at 50 °C, three phages (Shi22, Shi30 and Shi33) showed no significant differences at both temperatures, and one (AShi) showed a significant higher adsorption value at 50 °C. In addition, Shi40 and Shi113 showed adsorption values significantly higher at moderate temperatures, while Shi 22, Shi30 and Shi33 presented a significantly higher adsorption at extreme temperatures. To sum up, phages adsorbed efficiently at low (Shi3, Shi22, Shi30, Shi33, Shi34 and Shi88), at moderate (AShi, Shi34, Shi40, Shi93 and Shi113) and at high (AShi, Shi 22, Shi30 and Shi33) temperatures.

#### 3.2.3. Influence of Cations

**Figure 3:**
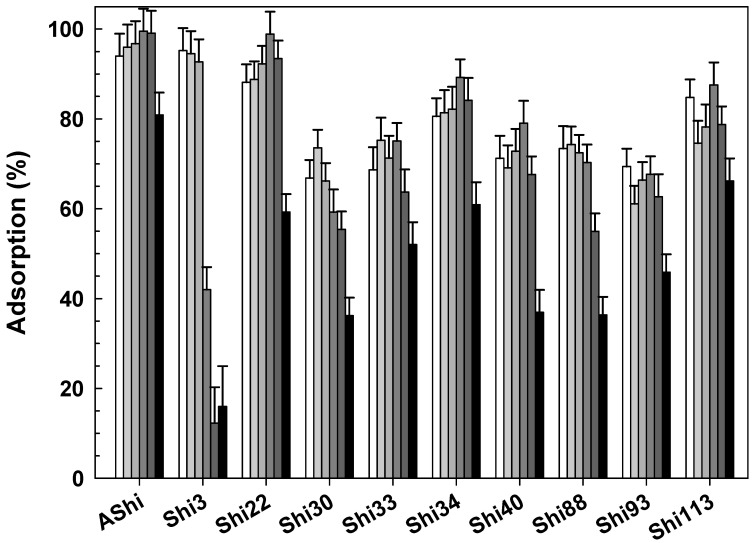
Phage adsorption in TSB at 25 °C (0.5%; ☐) with 1.0 g dL^−1^ (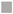), 2 g dL^−1^ (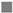), 4 g dL^−1^ (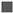), 10 g dL^−1^ (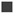) and 20 g dL^−1^ (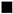) of Na^+^ on ATCC12022 viable cells. Values are the mean ± standard deviation (error bars) of three determinations.

Several cations, at a wide range of concentrations, are natural or added components of dairy and meat products. In addition, some of them are sometimes used by phages to adsorb on bacterial cells. When adsorption was evaluated in the presence of Na^+^ at different concentrations, eight phages adsorbed normally up to 10 g dL^−1^, while at 20 g dL^−1^, a significant reduction was observed for all the phages evaluated (Figure 3). Moreover, four out of these eight phages (AShi, Shi22, Shi33, Shi34 and Shi40) showed an increasing adsorption up to 4 g dL^−1^. In general, adsorption values ranged from 55 to 99% between 0.5 and 10 g dL^−1^ of Na^+^ depending on the phage analyzed. In addition, a large dispersion of values was observed when adsorption was compared among different phages. For instance, the minimum adsorption value achieved by AShi at 20 g dL^−1^ was higher than the maximum achieved by Shi30 at 1 g dL^−1^.

On the other hand, Shi3 and Shi88 were the two most affected phages since a significant reduction in their adsorption values was observed in treatments at 10 and 20 g dL^−1^ of Na^+^. Moreover, a significant decrease was observed for Shi3 at 4 g dL^−1^ where only 42 ± 5% of the particles were adsorbed to the cells, being the phage more affected at high concentrations (4, 10 and 20 g dL^−1^) of sodium.

Results indicate that adsorption was not significantly different for five out of the ten phages tested, namely AShi, Shi3, Shi22, Shi40 and Shi88, yet three of them (AShi, Shi22 and Shi88) showed a slightly increased adsorption at 1 mmol L^−1^ of Mg^2+^. Regarding phages Shi30, Shi33, Shi34 and Shi93, as the concentration of Mg^2+^ increases, adsorption decreased significantly, especially at the higher concentrations used (5 and 10 mmol L^−1^). Finally, Shi113 showed a significant reduction on particles adsorbed to *Shigella* cells, especially at the lowest (1 mmol L^−1^) and the highest (10 mmol L^−1^) concentrations assayed. However, adsorption was almost maximum (80 ± 4%) when compared with the control condition (82 ± 6%) without Mg^2+^ (data not shown). To sum up, phages showed their maximum adsorption between 2 to 4 g dL^−1^ of Na^+^ and from 0 to 1 mmol L^−1^ of Mg^+^.

#### 3.2.4. Influence of pH

**Figure 4:**
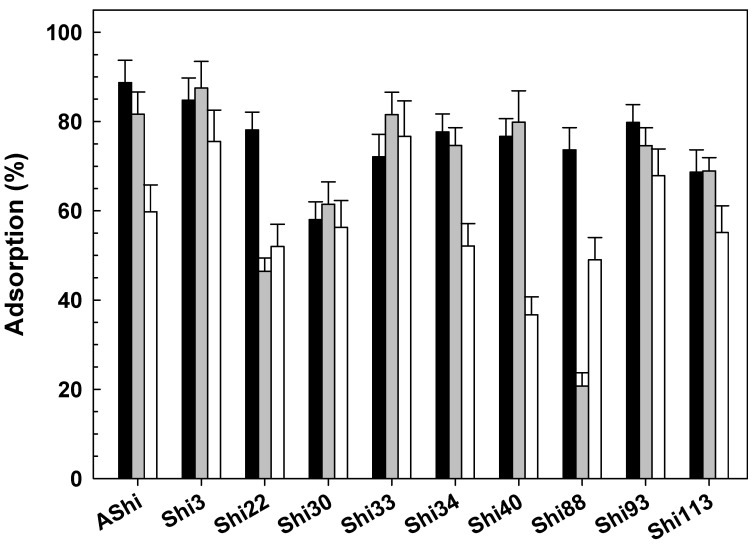
Phage adsorption in TSB at 25 °C and pH of 7.0 (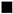; control), 4.0 (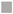) and 10.0 (☐) on ATCC12022 viable cells. Values are the mean ± standard deviation (error bars) of three determinations.

At pH 7, six bacteriophages (AShi, Shi22, Shi34, Shi88, Shi93 and113) exhibited adsorption values ≥ than at the extreme pH values (4 and 10) tested. Only four of them (AShi, Shi22, Shi88 and Shi93) showed a significantly higher adsorption at pH 7. For the other four phages (Shi3, Shi30, Shi33 and Shi40), the adsorption was ≥ at pH 4, yet only Shi33 showed a significant higher adsorption at the acidic pH than at the higher pH values assayed (7 and 10) (Figure 4). When the adsorption was compared between extreme pH values (4 and 10), eight out of the ten phages showed adsorption values ≥ at pH 4, though only six of them (AShi, Shi3, Shi34, Shi40, Shi93 and Shi113) showed a difference that was statistically significant. Conversely, the other two (Shi22 and Shi88) showed an adsorption significantly higher at pH 10. Phage viability losses were taken into account by calculating percentages regarding non-adsorbing controls at each pH evaluated.

#### 3.2.5. Influence of Sucrose

**Figure 5:**
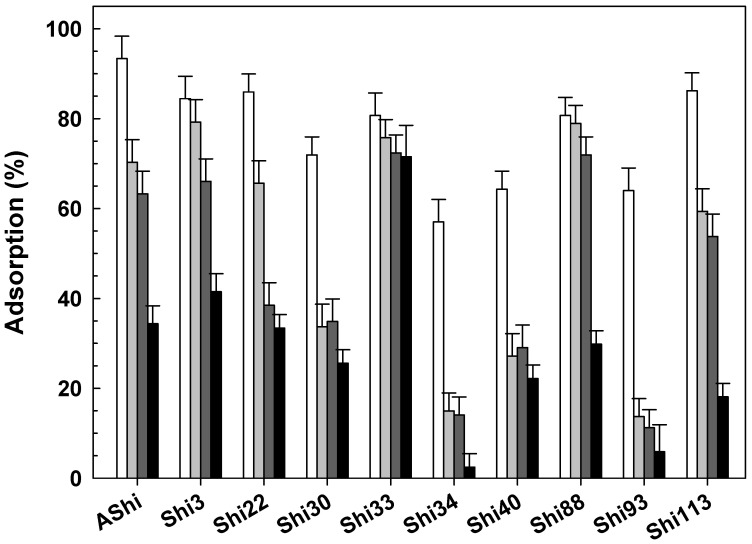
Phage adsorption in TSB at 25 °C without (☐) and with 5 g dL^−1^ (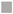), 10 g dL^−1^ (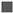) and 20 g dL^−1^ (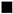) of sucrose on ATCC12022 viable cells. Values are the mean ± standard deviation (error bars) of three determinations.

Four phages showed that their adsorption significantly decreased as the concentration of sucrose increases (Figure 5). Specifically, AShi, Shi3, Shi22 and Shi113 exhibited moderate values ranging from ~40 to 70% at 10 g dL^−1^, while adsorption was significantly lower (18 to 41%) at 20 g dL^−1^. Shi34 and Shi93 presented the lowest adsorption of all the phages tested since their values ranged between 2.5 and 15%. In addition, the adsorption decreased sharply at the lowest concentration of sucrose (5 g dL^−1^) evaluated as well as for other two phages, namely Shi30 and Shi40. Adsorption achieved by Shi33 and Shi88 was significantly higher than for the other eight phages tested when challenged with sucrose. Both phages showed an adsorption to *Shigella* cells that ranged from 72 to 79%. Shi33 proved the higher adsorption value (72%) at the highest sucrose concentration assayed, while Shi88 exhibited a high reduction in the number of adsorbed particles (30%), especially at 20 g dL^−1^ of sucrose.

#### 3.2.6. Influence of Glycerol

**Figure 6:**
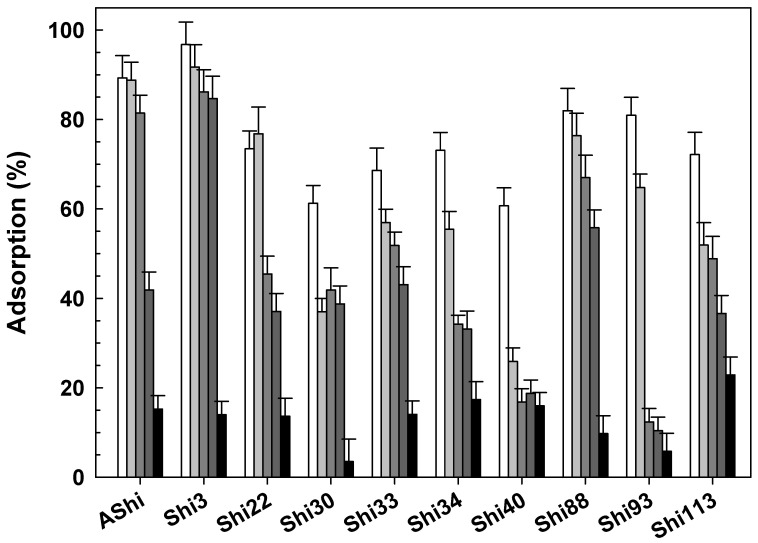
Phage adsorption in TSB at 25 °C without (☐) and with 5 g dL^−1^ (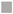), 10 g dL^−1^ (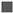), 20 g dL^−1^ (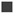) and 30 g dL^−1^ (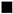) of glycerol on ATCC12022 viable cells. Values are the mean ± standard deviation (error bars) of three determinations.

Experiments showed that the adsorption significantly decreased as the concentration of glycerol increases (Figure 6). AShi, Shi3, Shi22, Shi88 and Shi113 were the more adsorbed phages, i.e., phages that exhibited the greater adsorption levels against increasing concentrations of glycerol. Among the 10 phages analyzed, Shi3 proved the highest adsorption value at 5 (92%), 10 (86%) and 20 g dL^−1^ (85%) of glycerol, whereas Shi113 exhibited the highest adsorption (23%) at the highest concentration of glycerol (30 g dL^−1^). On the other hand, Shi30, Shi40 and Shi93 were the least adsorbed phages since they were highly affected at high concentrations of glycerol, especially Shi30 at the highest concentration of glycerol evaluated.

#### 3.2.7. Adsorption in Meat

**Figure 7:**
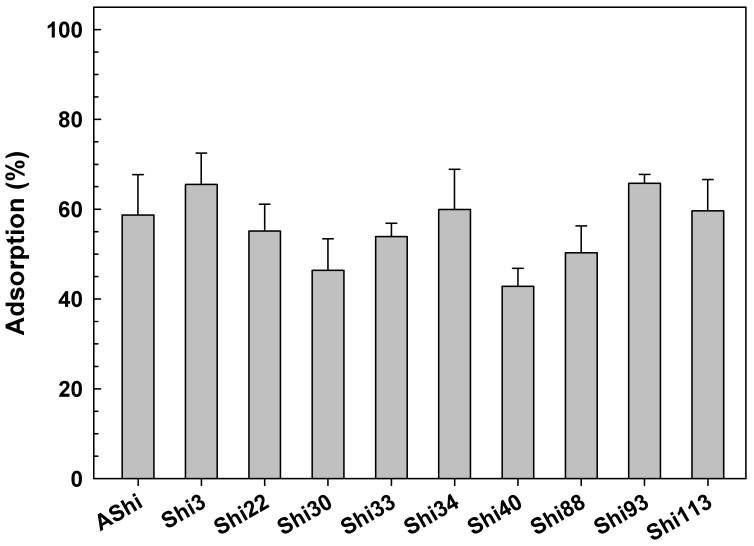
Phage adsorption in meat at 25 °C on ATCC12022 viable cells. Values are the mean ± standard deviation (error bars) of three determinations.

Adsorption experiments in food revealed that phages adsorbed differently to cells on the meat matrix. Figure 7 shows that Shi3, Shi 34 and Shi 93 were the most adsorbed phages, reaching an adsorption of 60 to 65% of their particles. Five phages (AShi, Shi22, Shi33, Shi88 and Shi113) showed values that ranged from 50 to 60% of their particles adsorbed in the food matrix, while, for the other two phages tested (Shi30 and Shi40), the adsorption was lower than 50%, namely 46 ± 7 and 43 ± 4%, respectively. The loss in the phage titer due to non-specific binding to meat was taken into account since percentages were calculated regarding the non-adsorbing controls.

## 4. Discussion

To determine if phages are useful with biocontrol purposes against pathogens, parameters such as burst size and latent period were studied through the one-step growth curve. A greater burst size (more phages) and a small latent period (phages generated quickly) are advantageous to kill foodborne pathogens. Results observed for *Shigella*-phages Shi22 and Shi30, namely high burst values with a large latent period, were similar to those found when Shahin and Bouzari investigated the burst size (53 PFU cell^−1^) and the latent period (20 min) of a phage (vB_SflS-ISF001) active against *Shigella flexneri* [18]. On the other hand, the other eight phages evaluated belong to another group with a lower burst size (14 to 17 PFU cell^−1^) (Table 1). Different burst values varying from 16 PFU cell^−1^ [19] to 151 PFU cell^−1^ [20] have been previously reported for phages of *Shigella*. In general, a high burst value leads to higher lytic activity [21], a wanted feature in phages that will be used in biocontrol applications. In agreement with our findings, similar results were observed by Xu and coworkers for the *Shigella* phage vB_ShiP-A7 (100 PFU cell^−1^, latent period of 35 min) [22] and by Ahamed et al. [23] for Sfin-1 (28 PFU cell^−1^, latent period of 5 min). On the contrary, several authors found phages with a high burst value and a short latent period such as phage vBSdyS-ISF003 (128 PFU cell^−1^, 10 min) [24] and phage 2019SD1 (151 PFU cell^−1^, 20 min) [20], and phages with a low burst value with a long latent period, e.g., phage pSf-2 (16 PFU cell^−1^, 30 min) [19] and SGF2 (38 PFU cell^−1^, 40 min) [25].

The first study carried out to characterize the adsorption process of the 10 *Shigella*-phages was the determination of the adsorption rate. Parameters such as the adsorption constant (*k*) need to be assessed to determine if these phages are suitable for biocontrol applications. Regarding the high adsorption observed for AShi and Shi22 (Table 1), a similar result was found for other phage where ~ 87% of the particles were adsorbed on the *S. flexneri* cells in 14 min [18].

From the experiments, we were able to calculate the adsorption rate constant (*k*) for each phage evaluated [17]. *K* values normally found in related phages such as T4 are around ~10^−9^ mL min^−1^ [26]. In accordance with our results, another author found that *k* values ranged between ~10^−11^ mL min^−1^ (phage M13) and ~10^−8^ mL min^−1^ (phage vB_ShiP-A7) [22]. In addition, it is important to highlight that the two phages with the highest *k* values (AShi and Shi22) were the only ones that achieved a high adsorption rate at the end of the experiments (17 min) with more than 96% of their particles adsorbed to ATCC12022 cells. Thus, taking into account that AShi and Shi22 adsorbed at a higher rate and in a greater proportion than the other phages evaluated, these particular phages have an advantage for potential biocontrol applications against the foodborne pathogen *S*. *flexneri*.

Adsorption of phages was evaluated at different temperatures of food interest. Foodstuffs are subjected to different temperatures during manufacture and storage. In addition, optimal growth temperatures for most pathogens, such as *Shigella flexneri*, are higher (37 °C), and most pathogens can survive long periods of time at 25 °C when the cold chain is lost. Therefore, it is important to assess phage adsorption to evaluate if they can be effective against pathogens in food environments at refrigeration and also at abusive temperatures. Prior to adsorption assays, viability of phages at refrigeration (4 °C), room (25 °C) and abusive (50 °C) temperatures was evaluated. All the phages tested showed a high resistance from 4 to 50 °C [14]. In agreement, two phages (vB_SflS-ISF001 and vB-SdyS-ISF003) active against *S*. *flexneri* [18] and *S*. *dysenteriae* [24], respectively, proved to have a high thermal stability in the range of -20 to 60 °C. Phages adsorbed efficiently at low (Shi3, Shi22, Shi30, Shi33, Shi34 and Shi88), at moderate (AShi, Shi34, Shi40, Shi93 and Shi113) and at high (AShi, Shi 22, Shi30 and Shi33) temperatures. Thus, a phage cocktail composed of the 10 phages evaluated in the present work covers a wide range of temperatures, a necessary and advantageous condition in biocontrol applications against foodborne pathogens. Furthermore, one of the strategies used to prevent the emergence of bacteriophage insensitive mutants is utilizing lytic phage cocktails since different phages from a given cocktail bind to different receptors on the bacterial cell surface.

No references were found regarding adsorption of *S. flexneri* phages at various temperatures. However, similar results were found for other foodborne pathogend, such as *S*. *dysenteriae*. Namely, an 80% [20] and 90% [24] of adsorption was found after 5 and 17 min of interaction at 37 °C, respectively. In addition, only in two studies was the adsorption rate for an *S. flexneri* phage determined. Shahin and Bouzari [18] found an adsorption of 87% after 14 min at 25 °C, while Xu and coworkers found an adsorption of 92% after a 5-min incubation with *S*. *flexneri* cells at 37 °C [22]. Although similar results were found by these two authors, only one temperature was assessed in both cases.

Several cations, at a wide range of concentrations, are natural or added components of dairy and meat products. Foods such as meat products possess variable concentrations of salt, reaching 4% or higher in some products, such as cold cuts. To determine whether bacteriophages can be useful on food environments, adsorption of the ten phages was assessed against increasing concentrations of Na^+^. Furthermore, phage adsorption against cations potentially involved in the adsorption process (Mg^2+^) was also evaluated. From previous studies, phages against *Staphylococcus aureus* [27] and *S*. *dysenteriae* [8] reported a significant increase in adsorption when Ca^2+^ was present. In addition, other authors found a high activity for vB_SflS-ISF001 (*Shigella flexneri* phage) when it was treated with increasing concentrations of Na^+^ [18], although adsorption of *S*. *flexneri* phages has not been previously evaluated against Na^+^. Furthermore, contrasting results were found for other phage of *Shigella* when it was tested against divalent cations such as Ca^2+^ [8]. However, a comparison could not be made regarding cations such as magnesium since no previous studies had been conducted.

Meat and dairy products have characteristic physicochemical parameters. After cattle slaughtering, meat pH falls to 5.5–6.0 while, during the milk fermentation process, the pH falls as low as 4.0–4.5. Therefore, it is important to evaluate the adsorption of phages in order to determine if they can be effective in these adverse conditions found in food environments. Our previous results indicated that phage viability was not significantly affected from pH 5 to pH 11, yet, at pH 3, a significant reduction in viability was observed. In addition, the activity of the 10 phages tested in this work was the same at 5 < pH < 9 and was slightly affected at 3 < pH < 5 and 9 < pH < 11 [14]. As previously observed by others [8,20,28], our phages were highly stable in a wide range of pH values (Figure 4). In addition, Shahin and Bouzari found a phage (vB_SflS-ISF001) active against *S*. *flexneri*, which proved to be stable in the range of 7 < pH < 10 [18]; however, assays have not been carried out to evaluate the adsorption at different pH values.

To determine the effect of different osmolarities present in foodstuffs such as dairy products (3 to 17 g dL^−1^) on the adsorption process, TSB with increasing concentrations of sucrose was prepared. Wang and coworkers [29] evaluated the inhibition of burst (adsorption, replication and burst) by sucrose and found that inhibition was achieved at a low osmolarity level (0.4 osmoles L^−1^). In our study, a similar value (0.15 osmoles L^−1^) was obtained when the inhibition of the adsorption process was evaluated. On the other hand, when the assays of sucrose and NaCl were compared, the inhibition achieved by sucrose is much greater than that observed with NaCl at the same osmolarity level. Namely, a significant inhibition of adsorption was achieved with sucrose at 0.15 osmoles L^−1^, while a similar inhibitory effect was observed for most phages at 3.42 osmoles L^−1^ when using NaCl. No previous studies related to the adsorption of *Shigella*-phages and sucrose could be found.

Regarding adsorption experiments with glycerol (Figure 6), as observed with sucrose, the adsorption process was significantly affected. The inhibition in the adsorption process was proportional to the number of the hydroxyl groups (OH) [29]. These groups (OH) are characteristics of alcohols, phenols and carboxylic acids, which are present in foods in a wide range of concentrations. Accordingly, other authors found that the concentration of glycerol was directly proportional to the inhibition of adsorption of an *S*. *flexneri* phage [29]. Glycerol proved a greater inhibition of the adsorption process than Na^+^ at a similar osmolarity level, although sucrose presented a similar inhibitory effect at a slightly lower osmolarity value than glycerol. Namely, adsorption was inhibited for most of our phages at 0.54 osmoles L^−1^ of glycerol, while a similar inhibition was achieved at 3.42 osmoles L^−1^ of Na^+^ and at 0.15 osmoles L^−1^ of sucrose. No recent studies related to the adsorption of *Shigella*-phages and glycerol could be found.

In a real food setting, phages showed a relatively high adsorption level since four to six (4–6) out of ten phage particles adsorbed to ATCC12022 cells in a solid food matrix such as meat. These adsorption values should be taken into account to calculate the correct number of particles per gram of food (PFU g^−1^) to achieve a successful biocontrol treatment. Biocontrol studies were conducted by other authors in which phages (ShigaShield^TM^) were used against *S. sonnei* strains in smoked salmon and yogurt [30]. However, no previous studies related to the adsorption of phages of *S. flexneri* in meat could be found.

## 5. Conclusions

To improve phage treatments against foodborne pathogens such as *S*. *flexneri*, adsorption studies at different conditions must be evaluated. The present study demonstrated that a significant amount of the initial number of phages was adsorbed to *S*. *flexneri* cells in a wide range of physicochemical conditions. Results suggest that our phages will be effective in controlling *S*. *flexneri* in food environments and in a food matrix such as meat. However, further studies are needed to improve the understanding of the adsorption process in the food matrix. In addition, sequencing and bioinformatics analyses are required to ensure that phages are safe for use as biocontrol of pathogens in food. Research previously conducted on *S*. *flexneri* phages was mostly focused on phage stability. To our knowledge, this is the first study focused on the adsorption process of *S*. *flexneri* phages against varying physicochemical conditions.

## Figures and Tables

**Table 1:** Parameters calculated from the one-step growth curve and adsorption rate experiments.

Phage	Burst Size (PFU Cell^−1^)	Latent Period (min)	Adsorption at 5 min (%)	Adsorption at the End (%)	Adsorption Constant (*k*) (mL min^−1^)
AShi	16	20	87 ± 9	99 ± 9	4.11 × 10^−9^
Shi3	17	15	70 ± 7	78 ± 8	2.43 × 10^−9^
Shi22	67	25	88 ± 8	96 ± 8	4.16 × 10^−9^
Shi30	64	30	56 ± 6	70 ± 7	1.66 × 10^−9^
Shi33	17	25	46 ± 5	56 ± 6	1.22 × 10^−9^
Shi34	14	20	49 ± 5	85 ± 8	1.35 × 10^−9^
Shi40	16	20	21 ± 3	64 ± 6	4.64 × 10^−10^
Shi88	15	25	45 ± 5	54 ± 5	1.19 × 10^−9^
Shi93	17	15	45 ± 5	60 ± 6	1.20 × 10^−9^
Shi113	15	15	76 ± 8	73 ± 7	2.85 × 10^−9^

PFU: plaque forming unit. Adsorption at 5 min (%): percentage of phage adsorption after 5 min interaction. Adsorption at 17 min (%): percentage of phage adsorption after 17 min interaction.

## Data Availability

The datasets generated during and/or analyzed during the current study are available from the corresponding author on reasonable request.

## Supplementary Materials

The following supporting information can be downloaded at: https://www.mdpi.com/article/10.3390/v14122815/s1, Figure S1: One-step growth curve in TSB at 37 °C on ATCC12022 viable cells. A representative curve (phage Shi22) is shown since a similar behavior was found for all the phages evaluated. Values are the mean ± standard deviation (error bars) of three determinations.
