# Supplementary material for: Influence of Physicochemical Factors on Adsorption of Ten Shigella flexneri Phages
Source: Viruses. 2022 Dec 16;14(12):2815. doi: 10.3390/v14122815 (PMC9788028; doi:10.3390/v14122815)
Supplement: Supplementary file 1 [file viruses-14-02815-s001.zip › viruses-2064858-supplementary.pdf]

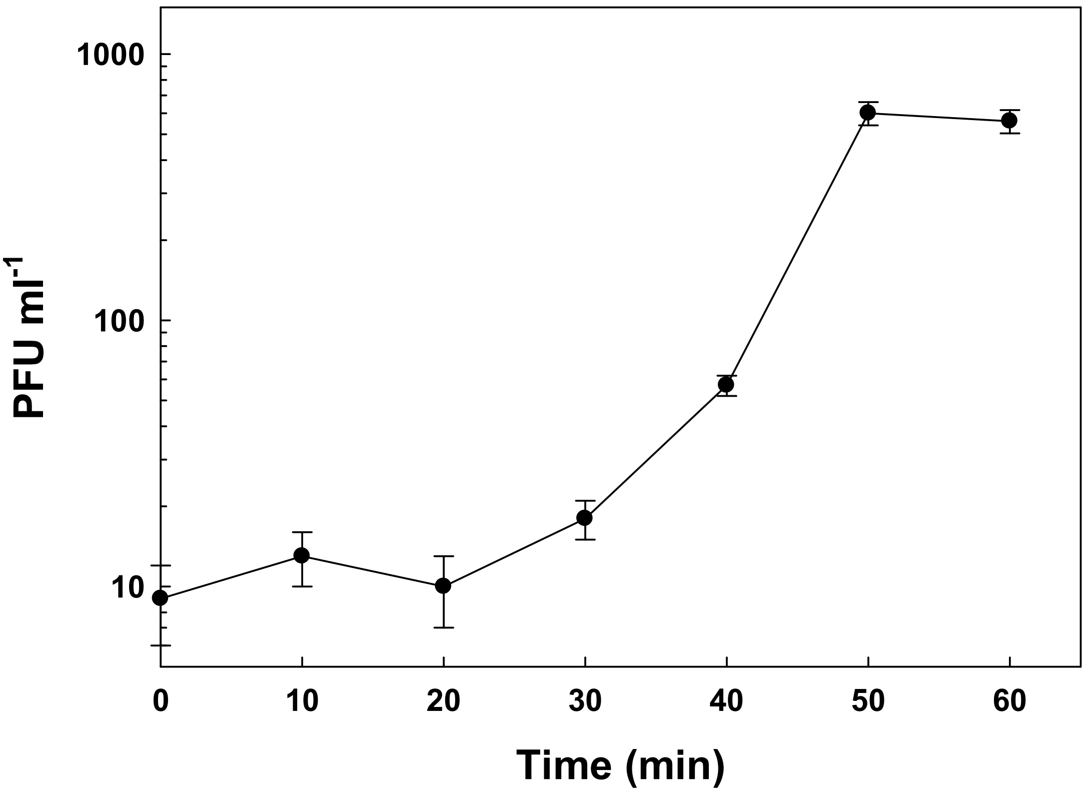

Figure S1. One-step growth curve in TSB at 37°C on ATCC12022 viable cells. A representative curve (phage Shi22) is shown since a similar behavior was found for all the phages evaluated. Values are the mean ± standard deviation (error bars) of three determinations.
